# Advancing prenatal diagnosis through comprehensive fetal cell‐free DNA screening

**DOI:** 10.1002/ctm2.70129

**Published:** 2024-12-22

**Authors:** Qiong Luo, Yanting Wu, Songchang Chen, Chenming Xu, Dan Zhang, Hefeng Huang, Jinglan Zhang

**Affiliations:** ^1^ Key Laboratory of Reproductive Genetics (Ministry of Education) and Department of Reproductive Endocrinology Women's Hospital Zhejiang University School of Medicine Hangzhou China; ^2^ Obstetrics and Gynecology Hospital Institute of Reproduction and Development Fudan University Shanghai China; ^3^ Institute of Medical Genetics and Development Zhejiang University Hangzhou China; ^4^ Shanghai Key Laboratory of Reproduction and Development Shanghai China

1

Over the past decade, prenatal screening of genetic conditions has witnessed transformative advancements, transitioning from imaging and biochemical techniques to molecular analyses, largely driven by the discovery of fetal cell‐free DNA (cfDNA) in maternal blood.^1^, ^2^ This breakthrough has revolutionized prenatal screening and diagnosis, enabling safer and more accessible detection of chromosomal abnormalities. However, significant challenges persist in addressing the full spectrum of genetic conditions with adequate accuracy, such as monogenic conditions and microdeletions/duplications, as well as sex chromosome aneuploidies. Traditional cfDNA screening primarily focuses on common autosome aneuploidies, such as trisomies 13, 18, and 21, but remains limited in identifying other genetic conditions. Furthermore, the detection of low‐level fetal DNA variants in maternal blood is impeded by the overwhelming presence of maternal DNA, compounded by the genetic heterogeneity and complexity of molecular etiologies underlying many fetal genetic conditions. Addressing these challenges, the recently developed “coordinative allele‐aware target‐enrichment sequencing” (COATE‐seq) technique represents a pivotal advancement.^3^ By facilitating the simultaneous detection of chromosomal abnormalities and monogenic conditions, COATE‐seq offers a unified, non‐invasive method for assessing fetal anomalies with diverse genetic etiologies. This technology employs high‐coverage, paired‐end sequencing to enhance the multidimensional analysis of sequencing read depth, allelic fraction, and linked single nucleotide polymorphisms, enabling accurate separation of the fetal genome from the maternal background. As a result, COATE‐seq achieves the sensitivity required to detect low‐level fetal variants.^3^ COATE‐seq leverages a targeted panel encompassing common aneuploidies, microdeletions, and genes associated with dominant conditions. Its efficacy was demonstrated in a prospective multicenter study involving 1090 high‐risk pregnancies recruited across three hospitals.^4^ Among these cases, 876 pregnancies exhibited fetal structural abnormalities identified via ultrasonography, providing a diverse cohort for evaluating COATE‐seq's performance in complex fetal anomalies. Blood samples collected after 12 weeks of gestation underwent COATE‐seq analysis, with findings validated through gold‐standard invasive prenatal or postnatal diagnostic techniques. COATE‐seq delivered impressive results, achieving a sensitivity of 98.5% and a specificity of 99.3%. Pathogenic genetic variants were identified in 135 pregnancies, marking a detection rate 60.7% higher than conventional cfDNA testing. Notably, these findings provide promising evidence that a comprehensive cfDNA‐based screening test can accurately detect fetal pathogenic variants at both chromosomal and single‐gene levels in high‐risk pregnancies through a noninvasive approach (Figure 1).

**FIGURE 1:**
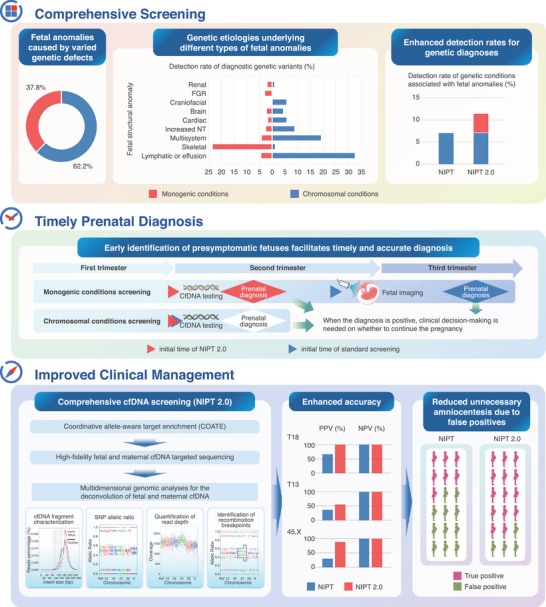
A comprehensive screening test of fetal cell‐free DNA. NIPT 2.0, a comprehensive screening test of fetal cell‐free DNA successfully detected pathogenic aneuploidies, microdeletions and monogenic variants linked to fetal anomalies, which resulted in a 60.7% increase in the detection rate for suspected fetal structural abnormalities. This test is based on COATE‐seq (coordinative allele‐aware target enrichment sequencing) which can be offered at early gestation which improves prenatal diagnosis and clinical management. NIPT: Non‐invasive prenatal testing; FGR, fetal growth restriction; NT, nuchal translucency; CfDNA, cell‐free DNA; PPV, positive predictive value; NPV, negative predictive value.

The comprehensive nature of COATE‐seq has profound clinical implications. By integrating the detection of monogenic conditions alongside chromosomal abnormalities, COATE‐seq significantly expands the diagnostic potential of cfDNA screening, allowing for earlier and more detailed risk assessments. These insights provide critical information for prenatal counseling and perinatal management, potentially improving outcomes for foetuses at risk of severe genetic conditions. For instance, in the study cohort, 23.5% of skeletal anomalies were linked to single‐gene variants, demonstrating the importance of monogenic condition screening.^4^ Pathogenic variants in genes such as *FGFR3*, *COL1A1*, *COL1A2*, and *PTPN11* are associated with severe developmental disorders that may not be detected during early gestation. By identifying such variants in these genes, COATE‐seq facilitates timely diagnostic follow‐ups and clinical decision‐making for optimal management.

While COATE‐seq offers a robust and innovative approach for managing pregnancies at risk for genetic conditions, several significant challenges must be overcome before it can achieve widespread implementation as a standard prenatal screening tool. Current research on COATE‐seq has largely concentrated on high‐risk pregnancies, leaving questions about the generalizability and diagnostic accuracy in low‐risk populations unanswered. To address this limitation, larger‐scale, multicenter studies that include diverse and varied obstetric cohorts are necessary. Such studies would provide data on its performance and reliability in routine prenatal care, thereby determining its potential applicability for broader clinical use. In addition, the detection of rare or complex genetic variants, such as large insertions, deletions, structural rearrangements, or pathogenic variants located in repetitive genomic regions, presents a significant challenge. These complexities may require further optimization of the COATE‐seq methodology or integration with complementary diagnostic tools or advanced computational algorithms, to enhance its accuracy. The adoption of COATE‐seq for expanded prenatal screen also raises critical ethical, social, and policy considerations, which magnifies concerns about equitable access, particularly given the high costs associated with high‐coverage sequencing. These costs could disproportionately limit availability to underserved and resource‐constrained populations. Addressing this issue will require targeted policy interventions, including subsidies or insurance coverage mandates, to make advanced prenatal screening universally accessible. Moreover, genetic screening requires robust counseling frameworks to support clinicians, parents, and families. The potential for any genetic findings necessitates thorough informed consent processes to ensure that individuals fully understand the scope, implications, and limitations of testing. As genetic data become more detailed and comprehensive, there is also a critical need to safeguard patient autonomy and confidentiality. Public health guidelines will need to be updated to reflect the broader scope of information provided by expanded fetal cfDNA screening. These updates should consider the ethical, social, and economic impacts of integrating advanced genetic technologies into standard prenatal care. Furthermore, policymakers must establish clear guidelines regarding the management of incidental findings and the storage, use, and sharing of genetic data to protect patient privacy and foster trust in these technologies.

Despite these challenges, COATE‐seq represents a transformative advancement in prenatal diagnostics. By extending the scope of non‐invasive screening to include both chromosomal abnormalities and monogenic conditions within a single test, it offers a comprehensive risk assessment platform that could redefine prenatal care. Its potential to identify severe genetic conditions early in pregnancy paves the way for more timely and targeted interventions, significantly improving maternal and fetal outcomes. However, realizing this potential requires coordinated efforts among researchers, clinicians, policymakers, and bioethicists. By expanding the capabilities of non‐invasive prenatal screening, COATE‐seq would advance maternal‐fetal medicine and establish new standards in prenatal care.

## CONFLICT OF INTEREST STATEMENT

The authors declare no conflicts of interest.
